# Laparoscopic Radical Total Gastrectomy and Pancreatosplenectomy for Synchronous Cancer of the Stomach and Pancreas

**DOI:** 10.7759/cureus.55927

**Published:** 2024-03-11

**Authors:** Motoki Ebihara, Kentoku Fujisawa, Shusuke Haruta, Hironori Uruga, Masaki Ueno

**Affiliations:** 1 Department of Gastroenterological Surgery, Toranomon Hospital, Tokyo, JPN; 2 Department of Pathology, Toranomon Hospital, Tokyo, JPN

**Keywords:** omental bursa, double cancer, pancreatic cancer, gastric cancer, laparoscopic surgery

## Abstract

The safety of laparoscopic surgery for advanced gastric and pancreatic cancers has been established individually, but there is little evidence for synchronous cancers. In this case, a 59-year-old man with a history of R-CHOP (rituximab, cyclophosphamide, doxorubicin, vincristine, and prednisolone) treatment for diffuse large B-cell lymphoma underwent laparoscopic surgery for a suspected pancreatic invasion of advanced gastric cancer. Pathology revealed double cancer of the stomach and pancreas.

Laparoscopic total gastrectomy and distal pancreatectomy were successfully performed. The patient had a pancreatic leak on postoperative day seven but was discharged from the hospital on postoperative day 21.

This case suggests the possibility of expanding the indications for laparoscopic surgery for similar cancers in the future. Additionally, the anatomical reticulum can be resected as a single mass using laparoscopy alone.

## Introduction

The safety of laparoscopic surgery for gastric or pancreatic cancer has been noted in various studies, but there is a lack of evidence for cases involving the invasion of other organs [1-6]. In 2022, the Japanese Laparoscopic Surgery Study Group (JLSSG) 0901 trial showed that laparoscopic surgery for advanced gastric cancer was non-inferior to open surgery in terms of five-year recurrence [7]. Lou et al. argue that for experienced surgeons, laparoscopic gastrectomy can be an alternative to open surgery [8]. In 2023, the DIPLOMA study showed that minimally invasive distal pancreatectomy was inferior to open distal pancreatectomy [6]. However, there are few reports on the safety and survival of laparoscopic surgery for double cancer.

In the present case, the possibility of pancreatic invasion by gastric cancer, gastric invasion by pancreatic cancer, or synchronous gastric and pancreatic cancers was considered. In either case, the preoperative strategy was to perform a total gastrectomy and distal pancreatectomy with en bloc resection of the tumor. We have previously reported that systematic mesogastric excision can be performed safely by laparoscopic surgery without dissection of the stomach or pancreatic mesentery [9].

## Case presentation

The patient was a 59-year-old male with a history of being a hepatitis B virus (HBV) carrier after R-CHOP (rituximab, cyclophosphamide, doxorubicin, vincristine, and prednisolone) treatment for diffuse large B-cell lymphoma (DLBCL). The patient was referred to our hospital after a submucosal lesion was detected during an upper endoscopy as part of a routine medical checkup. The patient was obese (height: 175.5 cm; weight: 108.5 kg; BMI: 35.2). Blood tests showed slightly elevated levels of liver enzymes (aspartate aminotransferase: 54 U/L; alanine aminotransferase: 59 U/L) but no other abnormalities. The tumor marker levels were within normal limits: carcinoembryonic antigen (CEA): 1.6 ng/mL; cancer antigen 19-9 (CA 19-9): 24 U/mL; alpha-fetoprotein (AFP): 9.2 ng/mL. Antibodies against *Helicobacter pylori* were elevated at 12 U/mL.

**Figure 1:**
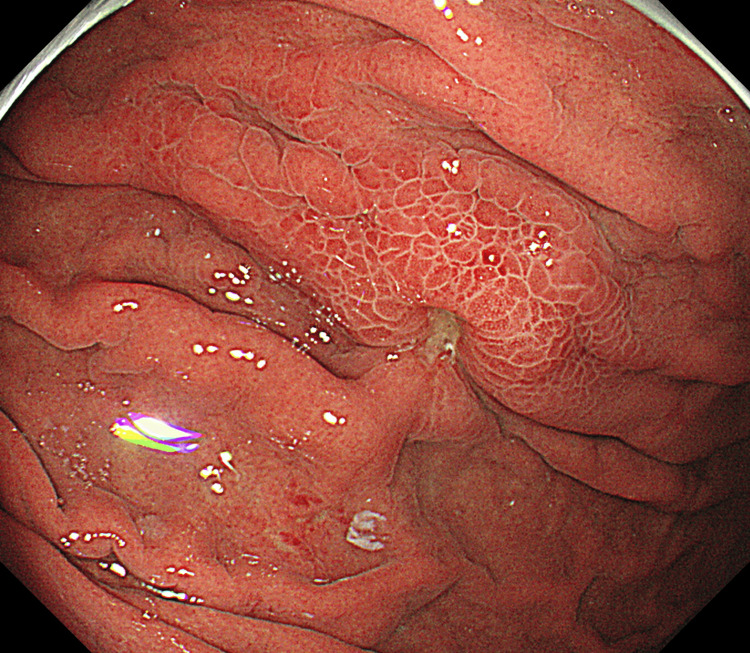
Preoperative upper endoscopic image Preoperative upper endoscopy reveals erythematous mucosa with small ulcers on the anterior wall of the upper gastric body of the greater curvature.

**Figure 2:**
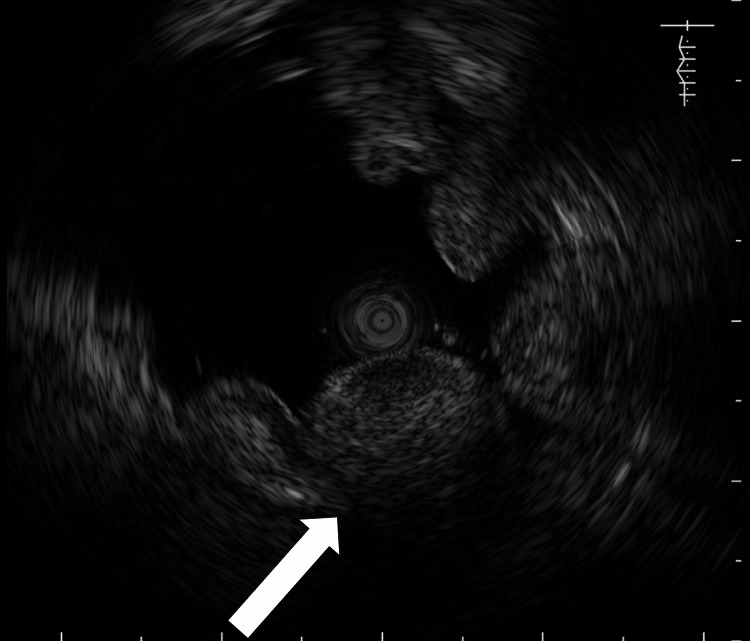
Preoperative ultrasound endoscopy Preoperative ultrasound endoscopy is diagnostic of advanced cancer of gastric submucosal cancer.

Preoperative upper endoscopy revealed erythematous mucosa with small ulcers on the anterior wall of the upper gastric body of the greater curvature (Figure 1). Ultrasound endoscopy showed wall thickening and a heterogeneous internal structure (Figure 2). Extramural observations and assessment of disease depth were difficult. The advanced carcinoma originated from the submucosa of the stomach, consistent with the initial diagnosis of skilled gastric carcinoma. Biopsy confirmed a diagnosis of poorly differentiated adenocarcinoma.

**Figure 3:**
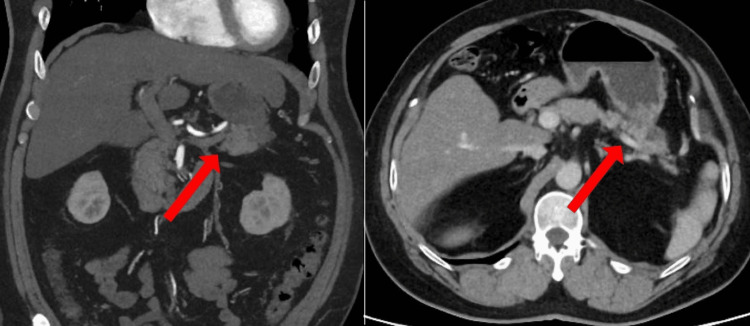
Preoperative computed tomography (CT) scan Preoperative CT scan showing a 37-mm tumor with suspected pancreatic invasion in the gastric body.

A computed tomography scan revealed a 37 mm-sized cyst and tumor with a substantial component in the pancreatic tail, indicating a possible invasion of gastric cancer into the pancreatic tail or pancreatic cancer invasion into the stomach (Figure 3).

**Figure 4:**
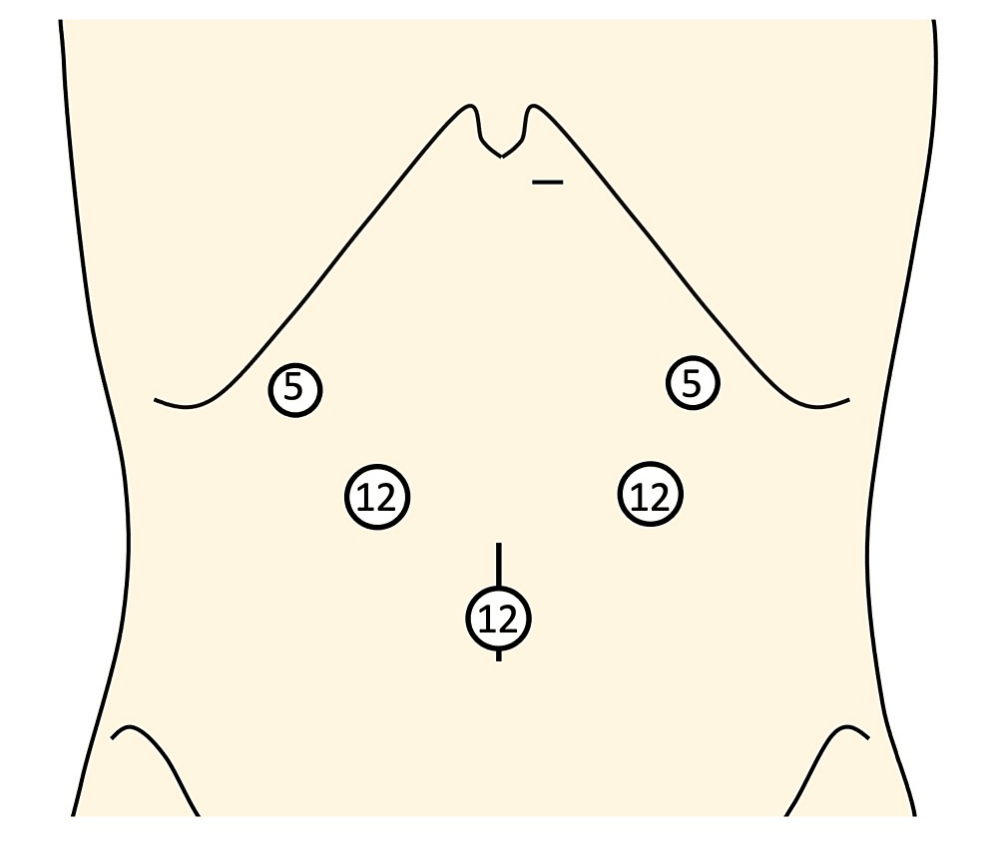
Port placement for laparoscopic surgery We performed a five-port laparoscopic procedure, as illustrated above. Image credits: Author.

Laparoscopic radical total gastrectomy, pancreatosplenectomy, lymph node dissection, and Roux-en-Y reconstruction were performed. Five abdominal ports were inserted, and surgery commenced (Figure 4). There was no intra-abdominal dissemination, and ascites cytology was negative.

**Figure 5:**
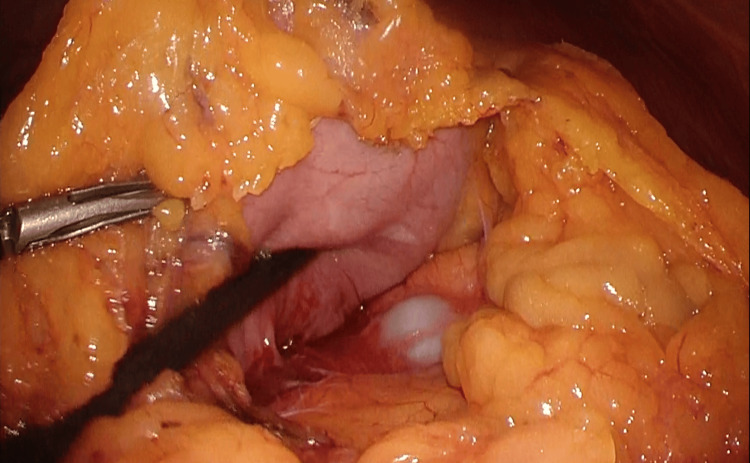
Intraoperative findings A white lesion is found in the pancreatic tail, which is diagnosed as a double cancer of the stomach and pancreas.

The greater omentum was dissected. When the omentum was dissected to the left and the omental bursa was opened, a hard tumor with white degeneration was found in the pancreatic tail, which was judged to be a synchronous cancer of the stomach and pancreas (Figure 5). We decided to perform total gastrectomy and distal pancreatosplenectomy.

The pancreas was extensively dissected dorsally at the Gerota's fascia layer connected to the suprapancreatic margin. At the suprapancreatic margin, the layer in front of the Gerota was extensively dissected using a medial approach and joined to the layer from the pancreatic inferior margin. By connecting this layer to the left and cephalic sides, almost total resection of the dorsal mesentery of the stomach could be performed. The operative time was six hours and 54 minutes, and the blood loss was 300 mL.

Postoperative fluoroscopy was conducted on the third postoperative day and confirmed that there were no issues at the anastomosis site. The patient began eating on the sixth day, and the drain at the anastomosis was removed on the seventh postoperative day. Although the patient developed a pancreatic fistula postoperatively, the overall postoperative course was favorable, and the pancreatic drain was removed on day 17.

**Figure 6:**
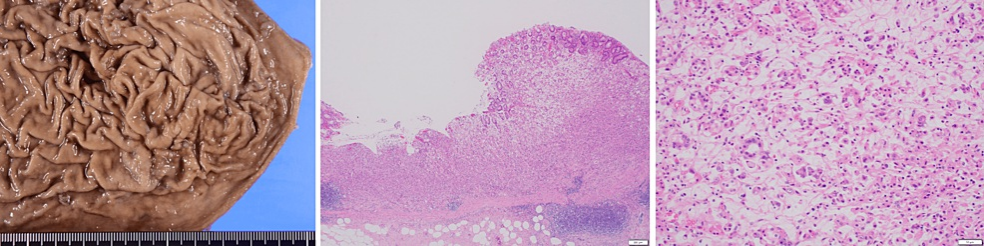
Pathological findings of the stomach The pathological diagnosis of the stomach is poorly differentiated adenocarcinoma. There are atypical cells with distinct nucleoli and enlarged nuclei: u/Gre, type 3, 29 × 22 mm, por+sig>tub2, pT3 (SS), pN0 (0/50).

**Figure 7:**
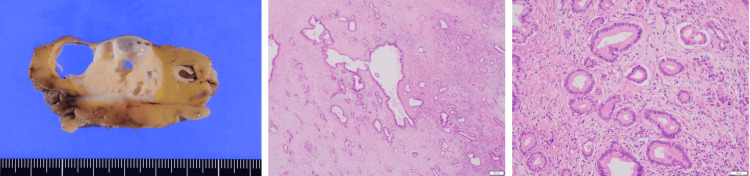
Pathological findings of the pancreas Atypical cells with enlarged nuclei and antacid cytoplasm present glandular luminal structures. The pathological diagnosis of the pancreas is invasive ductal carcinoma, well to moderately differentiated tubular adenocarcinoma: Pt, 41 × 27 × 22 mm, pT3, N1a.

Pathological examination revealed adenocarcinoma of the stomach: u/Gre, type 3, 29 × 22 mm, por+sig>tub2, pT3 (SS), pN0 (0/50) (Figure 6). It also revealed invasive ductal carcinoma of the pancreatic tail: Pt, 41 × 27 × 22 mm, pT3, N1a (Figure 7).

**Figure 8:**
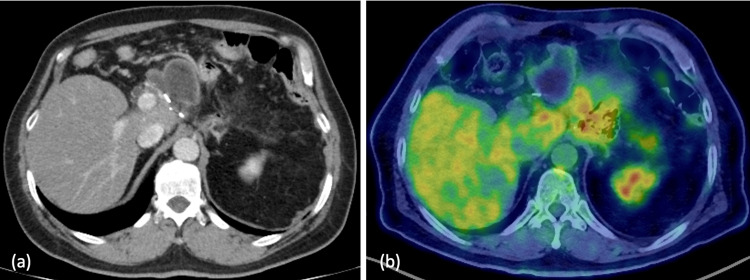
First postoperative CT scan and PET-CT scan (a) Subsequent postoperative follow-up CT revealed the presence of cystic lesions. (b) Subsequent PET-CT imaging indicated the absence of fluorodeoxyglucose (FDG) accumulation and the absence of postoperative recurrence.

Following discharge from the hospital, the patient received tegafur-gimeracil-oteracil-potassium (S-1) as a postoperative adjuvant chemotherapy regimen. Subsequent postoperative follow-up CT scan and positron emission tomography (PET)-CT scan revealed cystic changes, yet no evidence of recurrence was observed (Figure 8).

## Discussion

The eradication of *H. pylori* infection has led to a downward trend in the gastric cancer population. Gastric cancer remains the leading cause of malignancy in incidence worldwide. According to the GLOBOCAN data, gastric and pancreatic cancers ranked 5th and 12th, respectively. In terms of mortality rate, gastric and pancreatic cancer ranked 4th and 7th, respectively [10].

In the present case, three possibilities were raised preoperatively: synchronous cancer of the stomach and pancreas, pancreatic invasion of the stomach, and gastric invasion of the pancreas. In all cases, it is possible to resect the tumor en bloc, including the mesentery, by dissecting the gastric mesentery and pancreas [11]. Therefore, we decided to perform laparoscopic total gastrectomy plus pancreatosplenectomy and considered whether chemotherapy should be administered postoperatively, depending on the pathological diagnosis.

Various incidences of synchronous carcinomas in gastric cancer have been reported ranging from 1% to 10% [11-14]. Among these reports, cases of gastric cancer combined with pancreatic cancer are rare. In contrast, gastric cancer is the most common synchronous cancer associated with pancreatic cancer [15].

The advantages of resecting both tumors in a single surgery are that the tumor can be removed en bloc and that two surgeries may be more difficult due to adhesions. However, disadvantages include increased surgical difficulty and time.

A preliminary analysis of the long-term results of the JLSSG 0901 trial, a randomized phase 2/3 trial comparing the safety and curative efficacy of laparoscopic and open surgery for advanced gastric cancer, demonstrated that laparoscopic surgery was non-inferior in terms of five-year recurrence-free survival [1]. Laparoscopic and open surgeries have been compared worldwide, and laparoscopic surgery is now becoming the standard treatment [2]. In pancreatic surgery, the non-inferiority of laparoscopic surgery to open surgery for the resection of the pancreatic body tail has also been reported [3].

However, few studies have reported the safety or non-inferiority of laparoscopic surgery for multiple cancers. Currently, only case reports are available [4].

In this case, we performed laparoscopic total gastrectomy plus pancreatosplenectomy for double cancer of the stomach and pancreas. This meant that the omental bursa was removed en bloc. In other words, total laparoscopic resection of the stomach mesentery was performed.

The advantages of laparoscopic surgery include the ability to accurately identify layers through magnification, precise dissection, minimal invasiveness, and minimal blood loss due to insufflation. However, its disadvantages include a long operation time. To shorten the operating time and reduce the difficulty of the surgery, a skilled surgeon at a facility familiar with laparoscopy should perform laparoscopic double cancer surgery.

## Conclusions

Currently, there is insufficient evidence to substantiate its safety. Performing laparoscopic surgery for double cancer of the stomach and pancreas is difficult. However, we firmly believe that a skilled surgeon can safely perform this procedure. In the future, data should be collected jointly from multiple institutions. This will help establish evidence for the safety and curative potential of laparoscopic surgery.
